# Magnetic Nanoparticle Based Nonviral MicroRNA Delivery into Freshly Isolated CD105^+^ hMSCs

**DOI:** 10.1155/2014/197154

**Published:** 2014-03-31

**Authors:** Anna Schade, Paula Müller, Evgenya Delyagina, Natalia Voronina, Anna Skorska, Cornelia Lux, Gustav Steinhoff, Robert David

**Affiliations:** Department of Cardiac Surgery, Reference and Translation Center for Cardiac Stem Cell Therapy (RTC), University of Rostock, Schillingallee 35, 18057 Rostock, Germany

## Abstract

Genetic modifications of bone marrow derived human mesenchymal stem cells (hMSCs) using microRNAs (miRs) may be used to improve their therapeutic potential and enable innovative strategies in tissue regeneration. However, most of the studies use cultured hMSCs, although these can lose their stem cell characteristics during expansion. Therefore, we aimed to develop a nonviral miR carrier based on polyethylenimine (PEI) bound to magnetic nanoparticles (MNPs) for efficient miR delivery in freshly isolated hMSCs. MNP based transfection is preferable for genetic modifications *in vivo* due to improved selectivity, safety of delivery, and reduced side effects. Thus, in this study different miR/PEI and miR/PEI/MNP complex formulations were tested *in vitro* for uptake efficiency and cytotoxicity with respect to the influence of an external magnetic field. Afterwards, optimized magnetic complexes were selected and compared to commercially available magnetic vectors (Magnetofectamine, CombiMag). We found that all tested transfection reagents had high miR uptake rates (yielded over 60%) and no significant cytotoxic effects. Our work may become crucial for virus-free introduction of therapeutic miRs as well as other nucleic acids *in vivo*. Moreover, in the field of targeted stem cell therapy nucleic acid delivery prior to transplantation may allowfor initial cell modulation *in vitro*.

## 1. Introduction

Bone marrow derived human mesenchymal stem cells (hMSCs) have been shown to bear great potential for cell based therapeutic strategies. The ability of these cells to differentiate into various cell types and to secrete a large spectrum of antiapoptotic, angiogenic, and immunomodulatory factors offers the possibility to use them for tissue repair [1–4]. Furthermore, hMSCs are characterized by the expression of specific stem cell surface markers (e.g., CD29, CD44, CD73, and CD105) and the absence of hematopoietic markers (e.g., CD45, CD117) [1]. Moreover, it was shown that CD105 (endoglin) is a suitable surface marker for efficient purification of hMSCs from bone marrow [2]. Currently, several clinical trials, which involve hMSCs for the treatment of graft-versus-host disease, cartilage and meniscus repair, stroke, spinal cord injury, and Crohn's disease, are in progress [3]. Moreover, it has been recently shown that microRNA (miR) based genetic modifications of hMSCs before transplantation can significantly improve their therapeutic potential and survival rates [4–6]. Furthermore, miRs play an important role in stem cell regulation by influencing cell proliferation, differentiation, survival, apoptosis, and production of paracrine factors of hMSCs [4, 7, 8]. To date, several synthetic miRs are commercially available. However, miR delivery methods suitable for clinical applications will be crucial. Initially, viral carriers were widely used to transfer genetic material into target cells as they provide high transduction efficiency and long term gene expression. However, clinical applications of virus based gene carriers are limited as they may induce toxicity, immunogenicity, mutagenesis, and carcinogenesis [9, 10]. Thus, various nonviral methods were developed. Nonviral vectors have the benefit to be noninflammatory, noninfectious, and less toxic for efficient delivery of nucleic acids [9]. To date, numerous nonviral transfection carriers based on cationic lipids and cationic polymers are available on the market (lipoplexes and polyplexes, resp.). Thereof, Lipofectamine and polyethylenimine (PEI) are the best investigated nonviral transfection reagents for efficient nucleic acid transfer [11–13]. However, the clinical applications of Lipofectamine and PEI are restricted due to sensitivity and safety issues. Moreover, nonviral carriers can be combined with magnetic nanoparticles in order to improve selectivity and safety of delivery as well as to decrease side effects [14]. In 2002, Scherer and coworkers invented a novel technique termed “magnetofection.” This technique combines different well investigated gene delivery vectors (e.g., retrovirus, Lipofectamine, and PEI) with superparamagnetic iron oxide nanoparticles via salt-induced aggregation. The group showed that an externally applied magnetic field enhanced sedimentation of transfection complexes, thus improving transfection efficiency* in vitro* and* in vivo* [15]. In the last years, magnet based transfection (e.g., Magnetofectamine) has become a powerful tool for highly efficient and fast delivery of DNA [15, 16] as well as siRNA [17–19]. Our own group has developed a paramagnetic nonviral vector composed of nucleic acids condensed by biotinylated PEI and bound to streptavidin-coated iron oxide magnetic nanoparticles (MNPs) via biotin-streptavidin interactions. These MNP containing complexes, carrying therapeutic DNA, could be targeted by an external magnetic field to the site of interest* in vivo* [20]. Recently, we demonstrated that transfection with DNA/PEI/MNP complexes had a significantly higher transfection efficiency in cultivated hMSCs compared to DNA/PEI complexes even without the application of a magnetic field. We concluded a more rapid and efficient release of DNA from magnetic complexes compared to PEI polyplexes [21]. In contrast to DNA/PEI complexes, MNP containing complexes did not enter the nucleus due to strong biotin-streptavidin connections but released the DNA in the perinuclear region [14]. We have recently transferred this approach to transfection of hMSCs with miR, as the latter binds to its target mRNAs in the proximity of the nucleus.* In vitro*, we could demonstrate that miR/PEI/MNP complexes had a better long term silencing effect compared to mere miR/PEI polyplexes, which might be beneficial for clinical applications [22].

Although freshly isolated hMSCs are more relevant to clinical use, the quantity of these cells is too low to reach the desired effect [23, 24]. Thus, for our studies, as well as for clinical trials, hMSCs so far were expanded* in vitro* [21, 22, 25]. However,* in vitro* expansion of primary hMSCs is a costly and time-consuming procedure. In addition, the cells likely also lose their differentiation potential [2] and dramatically decrease their homing ability [26]. Therefore, genetic modifications of freshly isolated cells may be crucial to overcome these barriers and enable their clinical applications without previous* in vitro* expansion despite their low numbers available.

In this study, we applied a magnetic nonviral carrier for efficient miR transfection in freshly isolated hMSCs and compared it to commercially available magnetic vectors (Magnetofectamine, CombiMag particles) regarding uptake efficiency and cytotoxicity. We demonstrate that our novel magnetic transfection system is not inferior to the latter with respect to miR delivery and cellular tolerability.

## 2. Material and Methods

### 2.1. Isolation of CD105^+^ hMSCs

CD105^+^ cells were freshly isolated from sternal bone marrow. The bone marrow aspirates were obtained from patients during coronary artery bypass grafting at the Cardiac Surgery Department of the University of Rostock as previously described [27]. All donors gave their written consent to use their bone marrow for research proposes according to the Declaration of Helsinki.

At first, mononuclear cells (MNCs) were isolated by density gradient centrifugation. Afterwards, the CD105^+^ cell fraction was magnetically isolated using MACS technique according to the manufacturers' instructions (Miltenyi Biotec GmbH, Bergisch Gladbach, Germany). Briefly, 1 × 10^7^ MNCs were incubated with 20 *μ*L of CD105 MicroBeads (Miltenyi Biotec GmbH) for 30 minutes at 4°C. Next, suspension cells were washed with MACS buffer containing 2 mM EDTA (Gibco, Carlsbad, CA, USA), 0.5% bovine serum albumin (BSA, Sigma-Aldrich, St. Louis, MO, USA), and PBS. Subsequently, magnetically labeled cells were loaded onto a MS MACS column (Miltenyi Biotec GmbH) and placed in a magnetic field of a MiniMACS separator (Miltenyi Biotec GmbH). Afterwards, the positive CD105^+^ cell fraction was suspended in Mesenchymal Stem Cell Growth Medium (MSCGM, Lonza, Walkersville, MD, USA) containing 100 U/mL penicillin (PAA, Coelbe, Germany) and 100 *μ*g/mL streptomycin (PAA). Isolated CD105^+^ cells were immediately used in further* in vitro* experiments or expanded in MSCGM (Lonza) at 37°C and 5% CO_2_.

### 2.2. Immunophenotyping of CD105^+^ hMSCs

Cell surface markers of freshly isolated and cultured CD105^+^ hMSCs were fluorescently labeled with anti-human antibodies CD29-APC, CD44-PerCP-Cy5.5, CD45-V500, CD73-PE, CD117-PE-Cy7 (BD Biosciences, Heidelberg, Germany), and CD105-AlexaFluor488 (AbD Serotec, Kidlington, UK). Respective mouse isotype antibodies served as negative controls. 3 × 10^4^ events were acquired using BD FACS LSRII flow cytometer (BD Biosciences) and analyzed with BD FACSDiva Software 6 (BD Biosciences).

### 2.3. Functional Differentiation Assay of CD105^+^ hMSCs

Differentiation capacity of hMSCs was performed using the Human Mesenchymal Stem Cell Function Identification Kit (R&D Systems, Minneapolis, MN, USA) according to the manufacturers' protocol. After 20 days under differentiation conditions, fatty acid binding protein-4 (FABP-4) and osteocalcin for adipogenic and osteogenic differentiation were fluorescently labeled, respectively. Nuclei were counter stained with 4′,6-diamidino-2-phenylindol (DAPI, Invitrogen, Carlsbad, CA, USA). Samples were analyzed using ELYRA PS.1 LSM 780 microscope (Carl Zeiss, Jena, Germany) and ZEN2011 software (Carl Zeiss, Göttingen, Germany).

### 2.4. Preparation of Polyplex Based Transfection Complexes

For preparation of polyplex based transfection complexes (miR/PEI, miR/PEI/MNP, and miR/PEI/CombiMag complexes), Cy3 dye-Labeled Pre-miR Negative Control number 1 (Ambion, Austin, TX, USA) for monitoring uptake efficiency and cytotoxicity and Pre-miR miRNA Precursor Molecules Negative Control number 1 (Ambion) for testing complex formation were used. Branched polyethylenimine (MW = 25 kDa, Sigma-Aldrich) was biotinylated as described previously [22] and was stored in aliquots at 4.41 mM amine concentration at 4°C.

Initially, miR/PEI complexes with different molar ratios of PEI nitrogen and miR phosphate (N/P ratios) were prepared as previously described [28]. Briefly, miR and PEI were diluted in equal volumes of 5% glucose solution, mixed, and incubated for 30 minutes at room temperature.

In order to form miR/PEI/MNP complexes, Streptavidin Magnesphere Paramagnetic Particles (Promega, Madison, WI, USA) were sonicated and filtered using 450 nm Millix-HV PVDF syringe driven filter (Millipore, Tullagreen, Ireland). MNP filtrate was stored in aliquots at 4°C. Afterwards, 1 *μ*g/mL or 2 *μ*g/mL MNPs was added to miR/PEI complexes and incubated for 30 minutes at room temperature.

For miR/PEI/CombiMag complex formation, CombiMag reagent (OZ Biosciences, Marseille, France) was sonicated for 20 minutes. Afterwards, 0.025 *μ*L CombiMag per 1 pmol miR (0.025 *μ*L CombiMag/pmol) was mixed with miR/PEI complexes and incubated for 20 minutes at room temperature. All transfection complexes were freshly prepared before use.

### 2.5. Preparation of Lipoplex Based Transfection Complexes

For the formation of lipoplex based transfection complexes (miR/Magnetofectamine complexes), Cy3 dye-Labeled Pre-miR Negative Control number 1 (Ambion) was used for monitoring uptake efficiency and cytotoxicity.

At first, miR/Lipofectamine 2000 complexes were prepared. Therefore, miR and Lipofectamine 2000 transfection reagent (0.05 *μ*L Lipofectamine 2000/pmol miR, Invitrogen) were diluted separately each in 25 *μ*L of Opti-MEM I Reduced Serum Medium (Gibco) for 5 minutes at room temperature. Subsequently, miR and Lipofectamine 2000 solutions were mixed and incubated for 20 minutes at room temperature. For the formation of miR/Magnetofectamine complexes, CombiMag reagent (OZ Biosciences) was sonicated for 20 minutes. Afterwards, 0.025 *μ*L CombiMag/pmol was mixed with miR/Lipofectamine 2000 complexes as described above. Complexes were incubated for 20 minutes at room temperature according to Magnetofectamine instructions (OZ Biosciences). All transfection complexes were freshly prepared before use.

### 2.6. Condensation Assay of Transfection Complexes

The condensation of miR by PEI was studied by gel electrophoresis. miR/PEI complexes were prepared as described above, mixed with loading dye (Fermentas GmbH, St. Leon-Rot, Germany), and loaded onto 2% agarose gel containing ethidium bromide. An electric field of 100 V was applied for 30 minutes and image was taken using TS imaging system (Biometra GmbH, Göttingen, Germany).

### 2.7. Transfection

For transfection experiments, 1 × 10^5^ freshly isolated hMSCs per well were seeded in 48 well plates. Transfection complexes were prepared as described above and added drop by drop to the cells. Afterwards, cells were treated with and without the application of a magnetic field for 20 minutes using a Super Magnetic Plate (OZ Biosciences). Subsequently, cells were incubated for 24 hours at 37°C and 5% CO_2_.

### 2.8. Evaluation of Uptake Efficiency and Cytotoxicity

For quantification of uptake efficiency, hMSCs were transfected with complexes containing Cy3 dye-Labeled Pre-miR Negative Control number 1 (Ambion) as described above for 24 hours. To investigate cytotoxicity, cells were stained with Near-IR LIVE/DEAD Fixable Dead Cell Stain Kit (Molecular Probes, Eugene, OR, USA). Moreover, cells were labeled with Alexa Fluor 488 mouse anti-human CD105 (clone SN6, AbD Serotec) and fixed with 4% PFA. 3 × 10^4^ events were acquired using BD FACS LSRII flow cytometer (BD Biosciences) and analyzed with BD FACSDiva Software 6 (BD Biosciences). For determination of uptake efficiency, the number of living CD105^+^ Cy3-stained (Cy3^+^) cells in relation to the total cell number of living CD105^+^ cells was measured. To evaluate complex cytotoxicity, the percentage of dead CD105^+^ cells in relation to the total cell number of CD105^+^ cells was recorded.

### 2.9. Statistical Analysis

For all experiments, statistical analyses were performed by Student's *t*-test using SigmaPlot 11.0 software (Systat Software GmbH, Erkrath, Germany). Relative expression data of CD marker expressions are shown as mean ± standard deviation (SD). All other values are presented as mean ± standard error of the mean (SEM). A *P* value < 0.05 was considered to be statistically significant.

## 3. Results and Discussion

### 3.1. Results

#### 3.1.1. Characterization of CD105^+^ hMSCs

**Figure 1:**
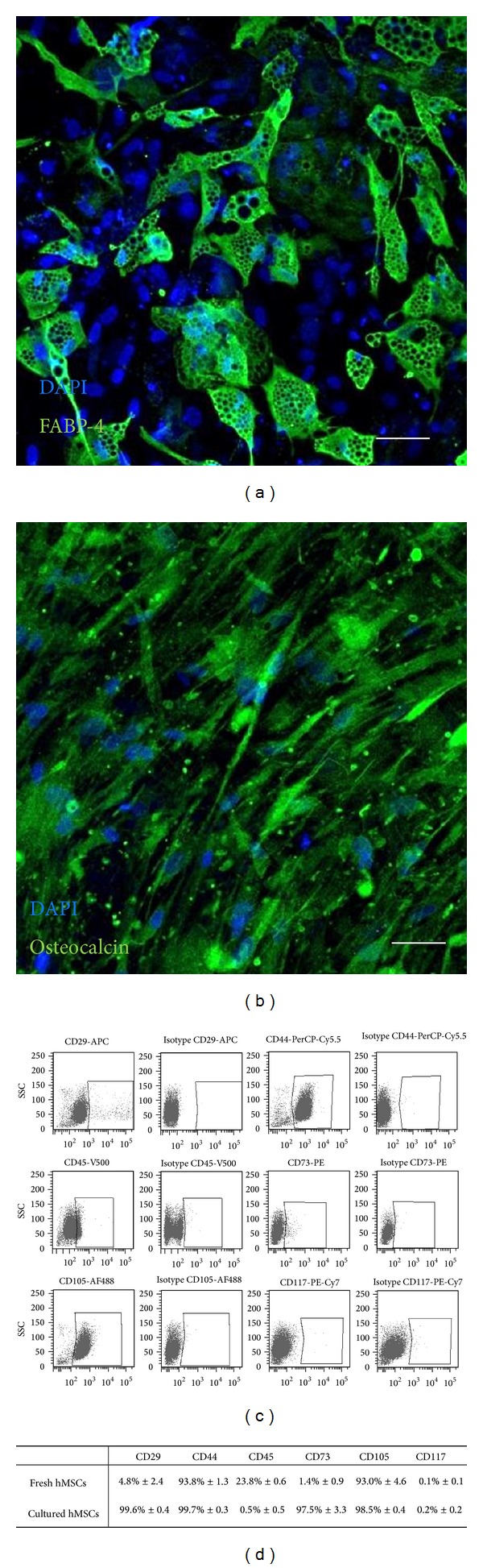
Characterization of CD105^+^ hMSCs. ((a), (b)) Functional differentiation capacity of freshly isolated CD105^+^ cells was shown by immunostaining of FABP-4 (green) for adipocytes (a) and osteocalcin (green) for osteocytes (b) after 20 days in appropriate differentiation medium. Nuclei were counter stained with DAPI (blue). Scale bars = 50 *μ*m. ((c), (d)) Immunophenotyping of freshly isolated ((c), (d)) and cultured CD105^+^ hMSCs (d) was evaluated by flow cytometry after staining of specific cell surface markers. Corresponding isotype controls were used as negative controls (c). The relative expression values of CD marker expression are shown as mean ± SD; *n* = 2 (d).

CD105^+^ hMSCs were isolated from bone marrow and immediately characterized by multilineage differentiation and specific surface marker expression before use in further experiments [29, 30]. To investigate the differentiation capacity of hMSCs, cells were cultivated in adipogenic and osteogenic differentiation medium, respectively, and examined by fluorescent microscopy.  Figure 1 illustrates that cells were able to differentiate into both adipocytes (Figure 1(a)) and osteocytes (Figure 1(b)). FACS analyses of freshly isolated CD105^+^ cells showed high expression of CD44 and CD105, a moderate expression of CD29 and CD45, and no expression of CD73 and CD117 (Figures 1(c) and 1(d)). Moreover, we compared the immunophenotype of freshly isolated cells with expanded CD105^+^ cells. Cultivated CD105^+^ hMSCs presented high expressions of stem cell markers CD29, CD44, CD73, and CD105 but had downregulated expression of hematopoietic markers CD45 and CD117 (Figure 1(d)).

#### 3.1.2. Characterization of Transfection Complexes

**Figure 2:**
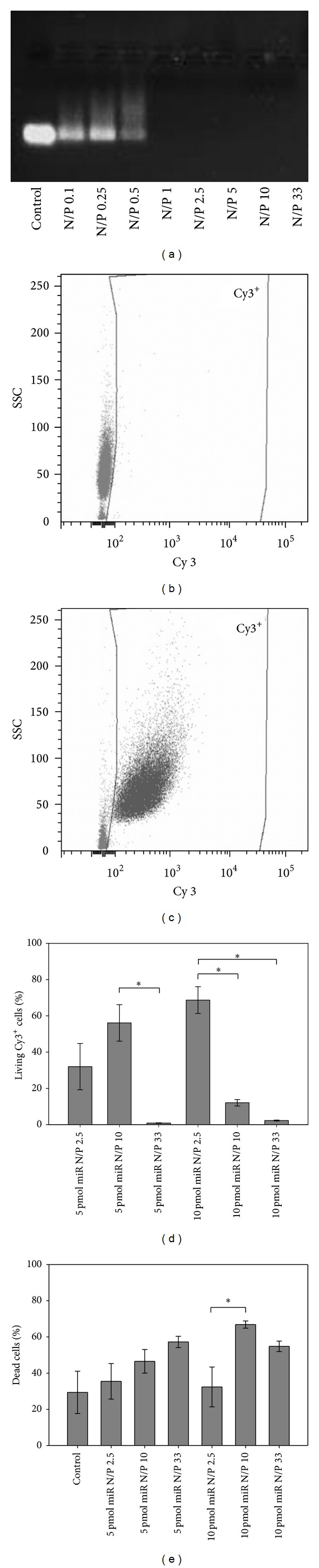
Characterization and transfection optimization of miR/PEI complexes. (a) Condensation of miR/PEI complexes composed of 20 pmol miR and different N/P ratios (ranging between 0.1 and 33) was analysed by gel electrophoresis. miR alone served as positive control. ((b), (c)) Gating strategy of FACS measurements. (b) Untransfected living CD105^+^ cells were used as negative control. (c) Living CD105^+^ cells transfected with Cy3-labeled complexes. ((d), (e)) Optimization of miR amounts and N/P ratios for transfection of freshly isolated hMSCs. hMSCs were transfected with Cy3-labeled miR/PEI complexes consisting of three different N/P ratios (N/P 2.5, N/P 10, and N/P 33) and two different miR amounts (5 and 10 pmol). 24 hours after transfection, uptake efficiency (d) and cytotoxicity (e) of complexes were analyzed by flow cytometry. Untransfected cells were used as control. Values are presented as mean ± SEM; *n* = 3; ∗ = *P* ≤ 0.05.

In order to examine condensation of miR by PEI, gel electrophoresis was performed. Thereby, different miR/PEI complexes with increasing amounts of PEI were investigated. Results demonstrated a big and sharp band for uncondensed miR that was used as a control (Figure 2(a)). At N/P ratios of 0.1, 0.25, and 0.5, miR has partly formed complexes with PEI. Due to the bigger size, miR/PEI complexes remained in the slots, while uncondensed miR migrated through the gel. However, the miR signal disappeared completely at N/P ratios greater than 1.

#### 3.1.3. Transfection Optimization of miR/PEI Complexes

In order to optimize transfection of freshly isolated CD105^+^ cells, polyplexes with different N/P ratios (N/P 2.5, N/P 10, and N/P 33) and miR amounts (5 pmol and 10 pmol) were tested using flow cytometry. Complexes with an N/P ratio of 10 combined with 5 pmol miR (56%) and an N/P ratio of 2.5 combined with 10 pmol miR (69%) showed the highest uptake efficiencies (Figure 2(d)). In order to increase uptake rates, higher N/P ratios (N/P 33) were investigated. However, an N/P ratio of 33 did not lead to further enhancement of uptake efficiencies. Likewise, potential cytotoxicity of the transfection complexes was investigated (Figure 2(e)). Thereby, untransfected cells were used as internal control (29% dead cells) which is reflecting cytotoxicity of the isolation procedure. Transfection complexes with an N/P ratio of 10 combined with 5 pmol miR led to moderately increased cell mortality (17%) compared to controls. Yet, polyplexes with an N/P ratio of 2.5 combined with 10 pmol miR showed no significant differences compared to controls (32% versus 29%). However, an increase in the N/P ratio (N/P 33) did lead to higher cytotoxicity in polyplexes with 5 pmol miR (57%) as well as with 10 pmol miR (55%). Therefore, with respect to the highest uptake rates and lowest cytotoxicity, polyplexes composed of an N/P ratio of 10 combined with 5 pmol miR and complexes with an N/P ratio of 2.5 combined with 10 pmol miR were considered to represent optimal compositions and were utilized in further experiments.

#### 3.1.4. Transfection Optimization of miR/PEI/MNP Complexes

**Figure 3:**
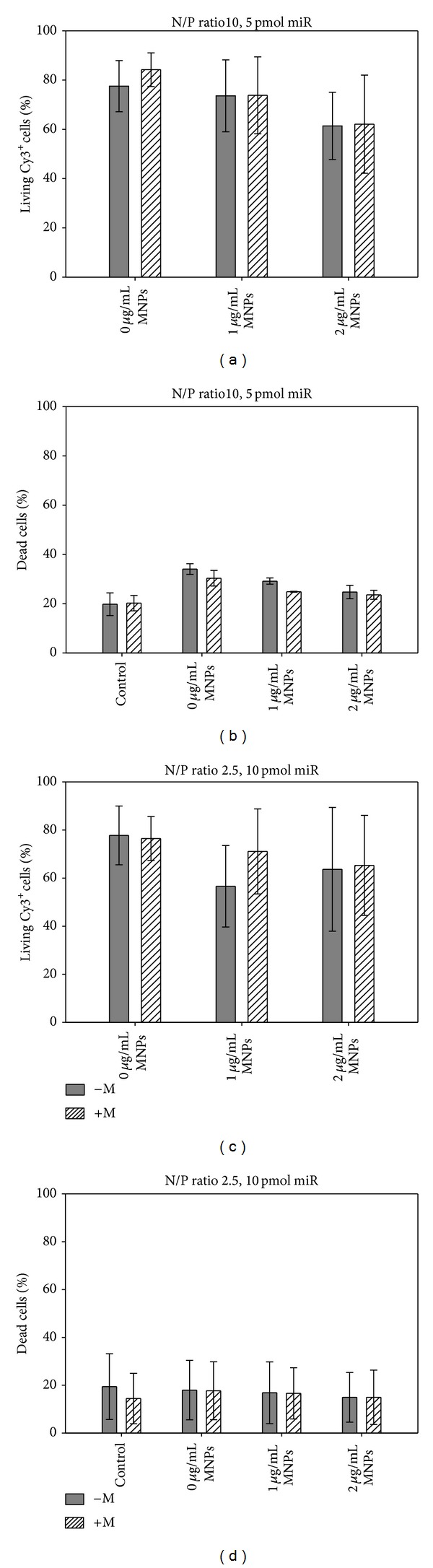
Transfection optimization of miR/PEI/MNP complexes. ((a)–(d)) Optimization of MNP amounts for transfection of freshly isolated hMSCs with (+M) and without (−M) the application of a magnetic field. hMSCs were transfected with Cy3-labeled miR/PEI and miR/PEI/MNP complexes composed of an N/P ratio of 10 combined with 5 pmol miR ((a), (b)) or an N/P ratio of 2.5 combined with 10 pmol miR ((c), (d)) and three different MNP amounts (0, 1, or 2 *μ*g/mL MNPs). 24 hours after transfection, uptake efficiency ((a), (c)) and cytotoxicity ((b), (d)) of complexes were analyzed by flow cytometry. Untransfected cells were used as control. Values are presented as mean ± SEM; *n* = 3.

In order to increase selectivity of the carrier and safety for clinical applications, the two optimized miR/PEI complexes were combined with different MNP amounts (1 or 2 *μ*g/mL MNPs). Therefore uptake rates (Figures 3(a) and 3(c)) and cytotoxicity (Figures 3(b) and 3(d)) of the different complex compositions as well as the influence of a magnetic field were investigated by flow cytometry. Magnetic polyplexes consisting of an N/P ratio of 10 combined with 5 pmol miR had similar uptake rates (up to 76%) compared to the corresponding miR/PEI complexes (81%) without the application of a magnetic field (Figure 3(a)). Moreover, cytotoxicity of these complexes was investigated. Magnetic polyplexes with 1 or 2 *μ*g/mL MNPs (27% versus 24%) showed no cytotoxic effect when compared to miR/PEI complexes (30%, Figure 3(b)). Likewise, polyplexes consisting of an N/P ratio of 2.5 combined with 10 pmol miR coupled to MNPs were investigated. Uptake rates of magnetic complexes with 1 or 2 *μ*g/mL MNPs (~65%), respectively, did not significantly differ compared to appropriate miR/PEI complexes (77%) in the absence of a magnetic field (Figure 3(c)). Furthermore, no significant differences in cytotoxicity were observed between the transfected cells (ranging from 15% to 18%) and the control (17%, Figure 3(d)). Additionally, the influence of a magnetic field was investigated. However, no significant improvement of uptake efficiency and reduction of cytotoxicity were observed when an external magnetic field was applied (Figure 3). For further experiments, two magnetic complexes were used: first an N/P ratio of 10 combined with 5 pmol miR bound to 1 *μ*g/mL MNPs and second an N/P ratio of 2.5 combined with 10 pmol miR bound to 1 *μ*g/mL MNPs.

#### 3.1.5. Comparison of miR/PEI/MNP Complexes to Established Transfection Reagents

**Figure 4:**
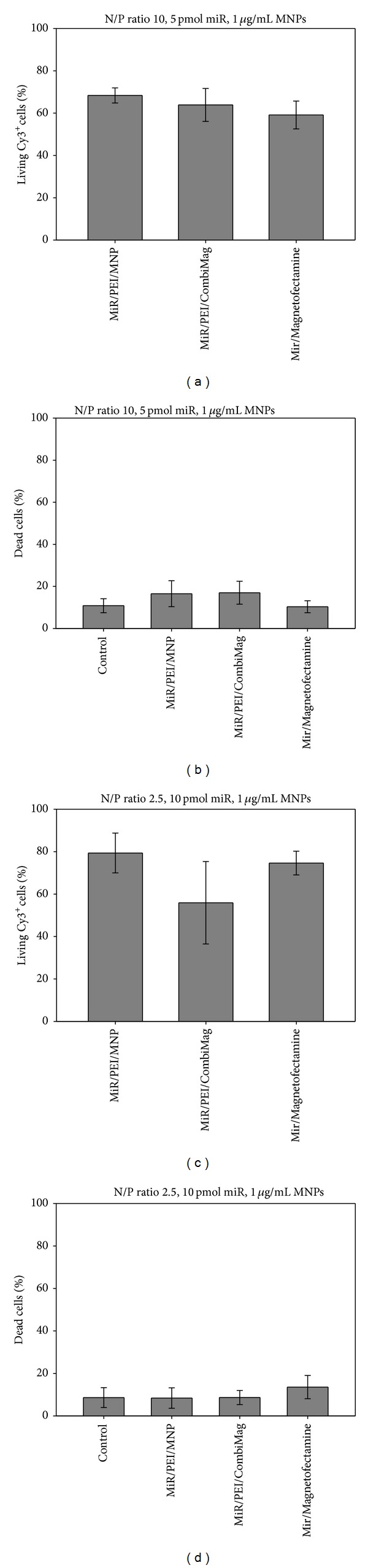
Comparison between optimized miR/PEI/MNP complexes and commercially available magnetic transfection reagents. Freshly isolated hMSCs were transfected with Cy3-labeled miR/PEI/MNP complexes composed of an N/P ratio of 10 combined with 5 pmol miR bound to 1 *μ*g/mL MNPs ((a), (b)) or an N/P ratio of 2.5 combined with 10 pmol miR bound to 1 *μ*g/mL MNPs ((c), (d)). For comparison with commercially available magnetic transfection complexes, appropriate amounts of Lipofectamine 2000 and CombiMag particles were used. 24 hours after transfection, uptake efficiency ((a), (c)) and cytotoxicity ((b), (d)) of complexes were determined by flow cytometry, while a magnetic field was applied. Untransfected cells were used as control. Values are presented as mean ± SEM; *n* = 3.

Optimized magnetic polyplexes were compared to commercially available magnetic vectors: Magnetofectamine and CombiMag particles. Therefore uptake efficiency (Figures 4(a) and 4(c)) and cytotoxicity (Figures 4(b) and 4(d)) of different transfection complexes were investigated using flow cytometry. miR/PEI/MNP complexes with an N/P ratio of 10 combined with 5 pmol miR bound to 1 *μ*g/mL MNPs showed high uptake rates (68%, Figure 4(a)). Moreover, no significant differences in uptake efficiency were observed between miR/PEI/CombiMag (64%) and miR/Magnetofectamine (59%) complexes. Mortality between treated and control cells was comparable and ranged between 11% and 17% (Figure 4(b)). Moreover, uptake efficiency and cytotoxicity of miR/PEI/MNP complexes composed of an N/P ratio of 2.5 combined with 10 pmol miR bound to 1 *μ*g/mL MNPs were investigated and compared to known transfection reagents. Uptake rates of miR/PEI/MNP complexes reached up to 79% and were compared to miR/PEI/CombiMag (56%) and miR/Magnetofectamine (75%, Figure 4(c)). Moreover, cell mortality of transfected cells (ranging from 9% to 14%) was not significantly different when compared to untreated control (9%, Figure 4(d)).

### 3.2. Discussion

Therapeutic potential of hMSCs can be improved by genetic modifications with distinct miRs [5, 6]. Our group has recently developed a magnetic nonviral transfection carrier consisting of biotinylated PEI bound to streptavidin-coated MNPs for highly efficient miR delivery (~75%) and applied it to cultivated hMSCs [22]. In general, hMSCs can be characterized by multilineage differentiation potential and the expression of specific surface markers (e.g., CD29, CD44, CD73, and CD105) [1]. Our previous studies have demonstrated that the purified CD105^+^ fraction of expanded hMSCs had a beneficial effect in cardiac tissue regeneration [27]. However, the time-consuming, expensive, and potentially detrimental cell expansion process might be avoidable as it has previously been described by Aslan et al. They tested CD105^+^ cells freshly isolated from bone marrow* in vitro* and* in vivo*. Importantly, these cells were indeed capable of giving rise to hMSCs bearing all typical properties after expansion [2]. Likewise in our study, freshly isolated CD105^+^ cells showed a slightly altered immunophenotype when compared to hMSCs after expansion (Figures 1(c) and 1(d)). However, our freshly isolated CD105^+^ cells also did adhere to plastic surfaces and showed a morphology and phenotype typical for hMSCs in expansion culture (Figure 1(d)). Moreover, we proofed the ability of these hMSCs derived from our freshly isolated CD105^+^ cells to differentiate into adipocytes (Figure 1(a)) and osteocytes (Figure 1(b)) under appropriate culture conditions. This clearly confirmed their stem cell character. Therefore, our findings are in line with the report of Aslan et al. [2].

To optimize miR transfection of freshly isolated CD105^+^ hMSCs, we initially tested miR/PEI complexes with different amounts of miR and PEI. In order to investigate the physicochemical properties of various miR/PEI complexes, condensation of miR by PEI was analyzed using gel electrophoresis (Figure 2(a)). Results demonstrated that, at N/P ratios from 1 to 33, no signals were observed. Therefore, we concluded that an N/P ratio 1 was sufficient to condense the whole amount of miR that is in correspondence with our previous findings [22]. Appropriate condensation of miR is essential for effective delivery into cells as it is protecting miR from early enzymatic degradation [11]. Furthermore, it prevents the activation of the innate immune system by double stranded RNA [31].

In order to establish efficient transfection complexes for miR delivery in freshly isolated CD105^+^ hMSCs, miR/PEI complexes with different N/P ratios (2.5, 10, and 33) and miR amounts (5 and 10 pmol) were tested (Figures 2(c) and 2(d)). Previous experiments showed that N/P ratios can significantly influence uptake efficiency of transfection complexes [32]; thus we selected 3 different N/P ratios for optimization experiments. Delyagina et al. showed that an N/P ratio of 2.5 showed the highest uptake efficiency of plasmid DNA in cultured hMSCs [21]. We have also demonstrated that miR was efficiently delivered using the same vector with N/P ratios of 10 and 33 [22]. Our current results demonstrated that miR/PEI complexes with 5 pmol provided the highest uptake efficiency at an N/P ratio of 10 (56%) for fresh hMSCs. In contrast to miR transfection in expanded hMSCs, we could further enhance miR uptake in freshly isolated hMSCs by increasing the miR amount to 10 pmol with an N/P ratio of 2.5 (69%). Due to the low N/P ratio, cytotoxicity could be decreased and was comparable to controls (32% versus 29%). The strategy of using low N/P ratios reduced the amount of used PEI that might be advantageous for genetic modifications. That is especially important for freshly isolated hMSCs as they might react very sensitive to potentially toxic reagents. Therefore, we decided to use miR/PEI complexes with an N/P ratio of 2.5 combined with 10 pmol miR for further experiments and compare it to complexes consisting of an N/P ratio of 10 combined with 5 pmol miR which were used in our previous work [22].

Furthermore, we combined optimized miR/PEI complexes with different MNP amounts to enable magnetic targeting of transfected cells (Figure 3). Based on previous work, miR/PEI/MNP complexes with 1 *μ*g/mL or 2 *μ*g/mL MNPs were investigated as they showed a significant increase in uptake efficiency compared to control (75% versus 50%) [22]. Our current investigations showed no significant differences in uptake rates and cytotoxicity of magnetic complexes with different compositions. The values of uptake efficiencies were comparable to those previously obtained for cultivated hMSCs [22] and were in the range from 56% to 79% (Figures 3(a) and 3(c)). Moreover, cell mortality was comparable to controls representing the basic level of cytotoxicity due to the isolation process (Figures 3(b) and 3(d)). Additionally, we investigated the influence of a magnetic field on miR transfection into freshly isolated cells. We observed no significant differences in uptake efficiency and cytotoxicity in cells transfected in the presence or absence of a magnetic field (Figure 3). These findings are in agreement with our previous data that showed efficient plasmid DNA transfection in hMSCs without the application of an external magnetic field [21]. This effect might be explained by the fact that a magnetic field has no influence on cellular uptake or intracellular transfection mechanism, as previously proposed [33].

Furthermore, we compared the performance of our miR/PEI/MNP vector and commercially available magnetic transfection reagents in freshly isolated CD105^+^ cells (Figure 4). Therefore we chose magnetic complexes consisting of an N/P ratio of 10 combined with 5 pmol miR bound to 1 *μ*g/mL MNPs as they showed good transfection values in both freshly isolated and cultured hMSCs [22]. Additionally, we selected complexes with an N/P ratio of 2.5 combined with 10 pmol miR bound to 1 *μ*g/mL MNPs as lower amount of PEI and MNPs in their composition facilitates their application. Magnetofectamine is a combined transfection reagent which consists of Lipofectamine 2000 and CombiMag particles and can be used for delivery of different nucleic acids in various cell types in the presence of an external magnetic field [34]. Lipofectamine is the most effective and best investigated cationic lipid, which can interact spontaneously with nucleic acids through electrostatic interactions forming stable lipoplexes [13, 35]. CombiMag particles can also serve as transfection reagent when combined with cationic polymers or cationic lipids [34]. However, the combination of CombiMag particles with Lipofectamine 2000 is proposed as the most effective approach. [34]. We could demonstrate that our miR/PEI/MNP vector was able to reach uptake rates similar to Magnetofectamine and miR/PEI/CombiMag complexes (Figures 4(a) and 4(c)). Moreover, cytotoxicity of all transfection complexes was comparable to controls indicating no cytotoxic effect of the different nonviral complexes on freshly isolated hMSCs (Figures 4(b) and 4(d)).

It has previously been shown by our group that both nonviral and viral magnetic complexes carrying labeled or therapeutic plasmid DNA can be targeted* in vivo * [20, 36]. Yet, thereby, no transfection into stem cells was performed. Furthermore, miR as nucleic acid of interest has so far not been used for* in vivo* targeting. This shortcoming led us to transfer our approach to an efficient delivery of miR into freshly isolated CD105^+^ hMSCs using three different magnetic carrier systems for the first time. All systems investigated yielded around 65% uptake of labeled miR combined with high cell viability. Therefore, we here introduce a novel carrier system which provides equal efficiency and cellular tolerance as commercially available magnetic transfection reagents.

Overall, the specific properties of the respective magnetic carriers for various applications may allow for direct nonviral delivery of therapeutic miRs as well as other nucleic acids* in vivo*. Moreover, introduction of these nucleic acids* in vitro* prior to cell transplantation may enable stem cell modulation for targeted stem cell therapy.

## 4. Conclusion

In this report, we successfully developed magnetic nonviral carriers for efficient miR transfection into freshly isolated CD105^+^ hMSCs. These magnetic vectors were able to reach high uptake rates (68% versus 79%) with no significant cytotoxic effect. The performance of the novel miR carrier equaled that of commercially available magnetic transfection reagents. Magnetic nanoparticle based miR transfection may become important to optimize stem cells meant for transplantation, although further preclinical experiments are required.
